# A Virus-Derived Immune Modulating Serpin Accelerates Wound Closure with Improved Collagen Remodeling

**DOI:** 10.3390/jcm8101626

**Published:** 2019-10-04

**Authors:** Liqiang Zhang, Jordan R. Yaron, Amanda M. Tafoya, Sarah E. Wallace, Jacquelyn Kilbourne, Shelley Haydel, Kaushal Rege, Grant McFadden, Alexandra R. Lucas

**Affiliations:** 1 Center for Personalized Diagnostics, Biodesign Institute, Arizona State University, Tempe, AZ 85287, USA; jyaron@asu.edu (J.R.Y.); agonza49@asu.edu (A.M.T.); 789sew@gmail.com (S.E.W.); Jacki.Kilbourne@asu.edu (J.K.); 2 Center for Bioelectronics and Biosensors, Biodesign Institute, Arizona State University, Tempe, AZ 85287, USA; Shelley.Haydel@asu.edu; 3 Chemical Engineering, Arizona State University, Tempe, AZ 85287, USA; Kaushal.Rege@asu.edu; 4 Center for Immunotherapy, Vaccines and Virotherapy, Biodesign Institute, Arizona State University, Tempe, AZ 85287, USA; grantmcf@asu.edu

**Keywords:** wound healing, serpin, immune modulation, scarring, hydrogel

## Abstract

Numerous treatments have been developed to promote wound healing based on current understandings of the healing process. Hemorrhaging, clotting, and associated inflammation regulate early wound healing. We investigated treatment with a virus-derived immune modulating serine protease inhibitor (SERPIN), Serp-1, which inhibits thrombolytic proteases and inflammation, in a mouse excisional wound model. Saline or recombinant Serp-1 were applied directly to wounds as single doses of 1 μg or 2 µg or as two 1 µg boluses. A chitosan-collagen hydrogel was also tested for Serp-1 delivery. Wound size was measured daily for 15 days and scarring assessed by Masson’s trichrome, Herovici’s staining, and immune cell dynamics and angiogenesis by immunohistochemistry. Serp-1 treatment significantly accelerated wound healing, but was blocked by urokinase-type plasminogen activator (uPAR) antibody. Repeated dosing at a lower concentration was more effective than single high-dose serpin. A single application of Serp-1-loaded chitosan-collagen hydrogel was as effective as repeated aqueous Serp-1 dosing. Serp-1 treatment of wounds increased arginase-1-expressing M2-polarized macrophage counts and periwound angiogenesis in the wound bed. Collagen staining also demonstrated that Serp-1 improves collagen maturation and organization at the wound site. Serp-1 has potential as a safe and effective immune modulating treatment that targets thrombolytic proteases, accelerating healing and reducing scar in deep cutaneous wounds.

## 1. Introduction

Large surface wounds, including lacerations and burns, are common and often complex injuries. In some cases, comorbidities such as diabetes and advanced age cause skin lesions to turn into nonhealing chronic wounds, reducing function and increasing risk of infection and bleeding. Chronic nonhealing wounds can be life threatening and are a major threat to public health with a large economic burden [1,2]. According to the NIH ARRA Impact Report (https://report.nih.gov), over 6 million cases of chronic wounds occur annually in the United States with a collective cost of more than $20 billion per year. Severe burn injuries cause about 40,000 hospitalizations and nearly 4000 deaths each year. Notably, these numbers do not include scar revisions, which amount to over 170,000 procedures annually in the USA [3]. There is thus a substantial need and growing interest in developing new treatments to improve wound healing [4,5,6,7,8].

The wound healing process is generally divided into three steps: (i) hemostasis and inflammation, (ii) new tissue generation, and (iii) remodeling [9,10]. Acute inflammation is central to healthy wound healing and is precisely regulated at both the cellular and molecular levels [11]. Conversely, sustained and excessive inflammation can exacerbate damage and result in chronic wounds [12,13]. Macrophages [14,15,16,17,18], neutrophils [19,20], and other cells of the innate immune system are crucial in initiating the early stages of wound healing via distinct and complex roles. For example, depleting macrophages during wound healing results in delayed re-epithelialization, dysregulated angiogenesis, and improper tissue remodeling [21]. Furthermore, macrophages exhibit the ability to polarize into functionally distinct subpopulations with differential roles in wound healing and promotion of anti-inflammatory M2 macrophages has been shown to accelerate cutaneous wound healing [22]. Neutrophils play an age-dependent role in wound healing, with depletion accelerating wound healing in young mice and delaying wound healing in aged mice [23,24]. Thus, the innate immune system is a critical and complex factor in controlling wound healing and represents a clinically relevant therapeutic target for improving wound healing.

In addition to cell-intrinsic activity, the circulating serine proteases in the coagulation and fibrinolytic cascades that regulate clotting and bleeding also coordinate the regulation of inflammation in wound healing [25]. For example, tissue-type or urokinase-type plasminogen activators (tPA or uPA) activate plasminogen to form plasmin in the fibrinolytic cascade, play key roles in the inflammatory stage of early wound healing, and are closely associated with angiogenesis [26]. Serine proteases of the coagulation and fibrinolytic cascade are regulated by serine protease inhibitors (SERPINs), which function by forming an irreversible, covalently linked suicide inhibition complex and permanently inactivating, targeted serine proteases [27]. As highly potent inhibitors of the serine proteases in these cascades, SERPINs also play a role in regulating wound healing [28,29]. Therapeutically, some groups have reported that application of recombinant SERPINSs (Alpha-1-antitrypsin, SERPINA1; anti-chymotrypsin, SERPINA3N) and related small molecules can promote wound healing [30,31,32,33,34].

**Figure 1:**
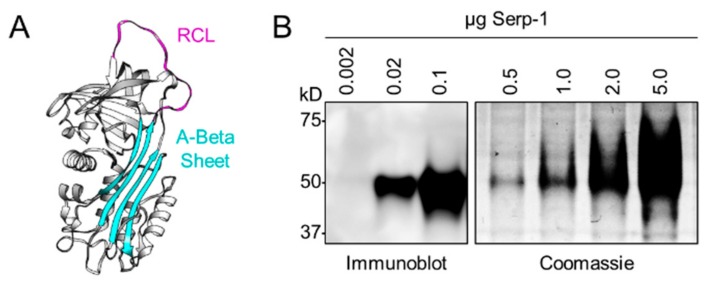
Myxoma virus-derived immune modulating serine protease inhibitor, Serp-1. (**A**) Structure of Serp-1 depicting a classic serpin structure with reactive center loop (RCL; magenta) and A-Beta sheet (cyan) indicated. (**B**) Immunoblot (left) and Coomassie (right) validation of purified recombinant cGMP Serp-1 using an anti-Serp-1 mouse monoclonal antibody.

Our group has investigated the therapeutic properties of Serp-1 in a broad range of inflammatory disorders, including transplant rejection and vascular disorders. Serp-1, is a 55 kDa secreted and glycosylated SERPIN derived from myxoma virus, a leporipoxvirus and the causative agent of myxomatosis in the European rabbit (*Oryctolagus cuniculus*). Myxoma virus has no pathogenicity in rodents or humans [35]. Serp-1 has a typical SERPIN structure with characteristic reactive center loop (RCL) and β-sheets (Figure 1A) [36]. In prior work, we systematically studied the therapeutic potential of recombinant Serp-1-mediated immune modulation in a wide variety of organisms, including humans, mice, rabbits, swine, and chickens [37,38,39,40,41,42,43]. Serp-1 is a first-in-class drug tested in Phase I and Phase IIa clinical trials in patients treated for unstable angina with coronary stent implantation [44]. In these patients, minimal-to-no adverse effects were observed with a major adverse cardiac event (MACE) score of zero and with minimal detectable neutralizing antibody. Further, Serp-1 was safe and effective in animal models of vascular injury and aortic, renal, and heart transplant [38,40,42]. Mechanistically, Serp-1 is a multipotent inhibitor of plasminogen activators, tPA and uPA, and complexes with the urokinase type plasminogen activator (uPAR), actin-binding protein filamin B, and vitronectin [30,31,45,46]. Serp-1 also binds and inhibits clotting factor X and thrombin (in the presence of heparin), providing a balanced effect on the thrombotic and thrombolytic cascades, thus reducing the risk of bleeding, and conversely, reducing the risk of excess clotting [43,47]. Interestingly, the targets of Serp-1 all have defined roles in wound healing, where they play central roles in modulating both coagulation pathway proteases, as well as inflammation, cell migration, wound closure, tissue remodeling, and fibrosis [48,49,50].

Here, we investigated the therapeutic potential of recombinant Serp-1 as a new biologic treatment approach for full-thickness excisional wounds in wildtype mice. Similar to other protein-based therapeutics, Serp-1 exhibits a relatively short half-life and complex stability profile [51]. It is therefore important to control Serp-1 protein release in order to extend the release time and increase stability for a long-term topical treatment, such as wound healing. Thus, we extended our study to examine sustainable delivery of Serp-1 in the wound bed with a chitosan-collagen biocompatible carrier.

## 2. Experimental Section

### 2.1. Proteins and Chemicals 

Purified, recombinant Serp-1 protein (m008.1L; NCBI Gene ID# 932146) was expressed and secreted by a Chinese hamster ovary (CHO) cell line (Viron Therapeutics Inc., London, ON, CA). GMP-compliant purification was performed by sequential column chromatographic separation with greater than 95% purity, as determined by Coomassie-stained SDS-PAGE and reverse-phase HPLC (Figure 1B). Serp-1 was endotoxin-free by LAL assay. Anti-Serp-1 mouse monoclonal antibody was provided by Viron Therapeutics (London, ON, CA). Type I collagen solution (C3867) and low molecular weight chitosan (448869l; 75–85% deacetylated) were purchased from Sigma-Aldrich (St. Louis, MO, USA).

All chemicals for trichrome staining, including phosphomolybdic/phosphotungstic acid solution, Biebrich scarlet solution 1%, aniline blue solution, Bouin’s fixative, acetic acid 1% aqueous, and Weigerts iron hematoxylin A and B, were from EMS (Electron Microscopy Sciences, Hatfield, PA, USA). Herovici’s collagen stain kit was from American MasterTech (Lodi, CA, USA). Hematoxylin and eosin for H&E stain were from Sigma-Aldrich. Information about each antibody used in this study is provided below when first mentioned.

### 2.2. Animals

All animal procedures in this study were approved by the Institutional Animal Care and Use Committee of Arizona State University under protocol #17-1549R. Male and female wild-type C57BL6/J mice aged 8–12 weeks were randomly selected and used in this study. Mice were kept on a standard 12/12 light-dark cycle in specific pathogen-free housing conditions and given food and water ad libitum. Mice were single-housed after the wounding procedure to prevent interference with wound healing. 

### 2.3. Wounding Surgery and Measurement

The splinted dermal wound healing mouse model was used for these studies, where the contraction exhibited in mouse skin is prevented by holding the wound open with a donut-shaped splint (Figure S1). Thus, wound closure due to contraction is excluded and observed healing is attributed to re-epithelialization. This method remains the most widely accepted and extensively used model to study skin wound healing in mice because non-splinted models do not accurately reproduce the granulation and re-epithelialization characteristics of human wound healing [52,53,54]. 

Mice were anesthetized by intraperitoneal injection of a cocktail of 120 mg/kg ketamine and 6 mg/kg xylazine, 0.1 mL/25 g bodyweight and prepped by shaving an area of approximately 1 × 1 inch spanning from between the ears to the peak of the spine, centered between each shoulder. The shaved area was sterilized by two washes with 2% chlorhexidine gluconate solution (Dyna-Hex 2^®^, Xttrium Laboratories, Mount Prospect, IL, USA) and 70% ethanol using cotton swabs.

A 3.5 mm punch biopsy was performed, centered in the shaved area to create the full-thickness wound excision, while preserving the panniculus carnosus beneath the skin. Treatment or control as a saline solution, or chitosan-collagen hydrogel preparation, were applied to the wound using a hand-held micropipette. In brief, 13 mice were given saline inoculation at the time of injury and 6 mice were given Serp-1 at the time of injury at doses of 1 or 2 µg. Six mice were given 1 µg of Serp-1 and followed by second bolus at 3 days post-wounding under isoflurane anesthesia without removing the splint. A second group of 6 mice were given Serp-1 in chitosan-collagen hydrogel treatment applied at the time of injury and 6 mice were given chitosan-collagen hydrogel alone. Another cohort of 10 mice were locally given 1 μg of anti-uPAR antibody (R&D Systems, AF534, Minneapolis, MN, USA) in 10 μL saline and 5 of them were given 1 μg of Serp-1 in 10 μL saline after the antibody solution was absorbed, while the rest, 5 mice, were given 10 μL saline as control group. All antibody-treated mice were given either second bolus of antibody/Serp-1 combination or antibody alone at 3 days post-wounding. 

A donut-shaped silicon splint (O.D. 15 mm; I.D. 5.0 mm; Culture-Well™, Grace Bio-labs, Bend, OR, USA) with Tegaderm™ (3M Company, Maplewood, MN, USA) affixed to one side was coated with cyanoacrylate glue (Krazy Glue^®^, High Point, NC, USA) on the opposite side and carefully placed on the back of the mouse with the biopsy site centered within the inner hole. Six interrupted sutures (4-0 black Ethilon monofilament with a FS-2 reverse cutting needle; Ethicon, Inc., Somerville, NJ, USA) were placed around the outer edge of the splint to complete the procedure. Mice were allowed to recover and returned to single-housed cages for the remainder of the experiment. Mice were anesthetized by 1–3% isoflurane at day 7 post-wounding, splints were carefully removed to prevent secondary damage caused by self-removal, and then mice were returned to single-housed caging. 

On the day of the procedure (Day 0) and on every subsequent day of follow-up, for a total of 15 days, mice were collected and wounds assessed while awake. Digital images were collected. Planimetric measurements of wound healing progress was performed in ImageJ/FIJI (NIH, Bethesda, MD, USA) and calibrated to known pixel-to-size measurements [55].

### 2.4. H&E and Immunohistochemistry

Skin tissues were collected at day 1, 4, 7, and 15 and fixed in 10% neutral-buffered formalin for at least two days before tissue processing with a Leica TP1050 and embedding in paraffin with a Leica EG1160 embedding station. Blocks were serially sectioned using a Leica RM2165 microtome (4 μm sections) and stained with hematoxylin and eosin (H&E) by standard procedure. Sections were additionally stained by immunohistochemistry (IHC) for CD31 (Abcam, Cambridge, UK, #ab28364, 1:100), Ly6G (Invitrogen, Carlsbad, CA, USA, #14-5931-82, 1:100) and arginase-1 (Cell Signaling, Danvers, MA, USA, #93668, 1:200), and with Masson’s trichrome [56] and Herovici’s polychrome [57] as special stains for collagen. 

### 2.5. Pathology Imaging and Analysis

Slides were imaged on an Olympus BX51 upright microscope equipped with an Olympus DP74 color CMOS high-resolution camera operated by cellSens Dimensions v1.16. Images (Olympus, Waltham, MA, USA) were collected as objective-calibrated TIFFs and subsequently analyzed and processed in ImageJ/FIJI [55]. Positively stained cells were counted per high power field for each treatment group. 

Quantitative collagen texture analysis was performed in ImageJ/FIJI [55]. Briefly, images were deconvoluted with the plugin “Colour Deconvolution 1.7” using the methods described by Ruifrok and Johnston [58]. Local thickness of collagen bundles was determined with the plugin “LocalThickness 4.0.2” using the methods described by Saito and Toriwaki, and by Hildebrand and Rüegsegger [59,60]. Regularity of collagen bundles was determined with the plugin “Directionality”, as previously reported [61].

### 2.6. Enzyme-Linked Immunosorbent Assay (ELISA)

Wound beds and surrounding tissue (same total size) were collected on days 1, 4 and 7 post-wounding and homogenized with a blade homogenizer into 400 µL RIPA buffer containing 1× protease inhibitor cocktail (Bimake, Houston, TX, USA, #B14001) on ice. Tissues were rotated at 4 °C for 2 hours, centrifuged at 14,000× *g* for 15 minutes at 4 °C and supernatant was transferred to a new tube. Vascular endothelial growth factor (VEGF) was quantified with the Mouse VEGF DuoSet ELISA (R&D Systems, Minneapolis, MN, USA, #DY493) and DuoSet ELISA Ancillary Reagent Kit 2 (R&D Systems, Minneapolis, MN, USA, #DY008) according to manufacturer’s instructions. Quantified VEGF was normalized to total protein using the BCA assay (ThermoFisher Scientific, Waltham, MA, USA, #23225).

### 2.7. Preparation and Characterization of Chitosan-Collagen Hydrogels with and without Serp-1

The procedure for preparing the chitosan-collagen hydrogel was adapted from a previously published method [53]. Low molecular weight chitosan was swollen by adding 10 mg chitosan to 10 mL of deionized water and rotating overnight at 4 °C. The excess water was removed from the mixture by centrifugation at 1000× *g* for 15 min and the swollen chitosan product (as a partial suspension) was frozen at −20 °C for 8 hours followed by incubation overnight at 4 °C. Serp-1 (30 µg in 16 µL) was added and the mixture was rotated at 4 °C for 8 hours and then lyophilized overnight. Shortly (less than 1 hour) before treatment or in vitro assays, the lyophilized product was added to Type I collagen solution (Sigma Aldrich Life Science, St. Louis, MO, USA, C3867) to a total volume of 300 µL to form a chitosan-collagen/Serp-1 hydrogel at a concentration of 1.0 µg Serp-1 per 10 µL gel. Chitosan-collagen hydrogel without Serp-1 (saline only) was used as a control. The final product had well-characterized biodegradation in the skin and other tissues [62].

For scanning electron microscopy analysis, chitosan-collagen hydrogels were fixed in 2% glutaraldehyde at room temperature for 15 minutes. Fixed hydrogels were washed 3x in deionized water for 10 minutes each. Washed hydrogels were dehydrated in a graded ethanol series (30%, 50%, 75%, 95%, and 3x 100% anhydrous) at room temperature for 10 minutes each. Dehydrated hydrogels were then critical point-dried using liquid CO_2_ as the transition fluid in a Balzers CPD-020 drying apparatus. Samples were then mounted on AI stubs and sputter-coated with gold for 5 minutes at 8 mA current in a Technics Hummer-II sputter coater, resulting in a coating of approximately 10 nm thickness. Samples were then imaged in a JEOL 6300 SEM operated at 15 kV with images acquired with an IXRF Systems Model 500 digital processor.

To test protein release from hydrogels, three preparations were made containing 0, 1.0, and 3.0 µg Serp-1 per 10 µL gel, as mentioned above. Thirty microliters of gel aliquot per well were loaded into a 96-well plate, 4 wells for each gel. Two hundred microliters of saline containing 0.01% (w/v) sodium azide were added to each well and incubated at 37 °C. At each designated time point, 20 µL of the incubating solution was collected from each well, followed by adding 20 µL of fresh saline back into the same well. At day 4, gels were boiled with 200 µL of 1× reducing Laemmli buffer after completely removing liquid from the wells. Standard Western blotting was performed for Serp-1 detection using a monoclonal anti-Serp-1 antibody (gift from Viron Therapeutics Inc., London, ON, CA, AQH9.6).

### 2.8. Statistics

Statistical significance analysis was performed by analysis of variance (ANOVA) and Student’s T-test using GraphPad Prism v8.2.1 (GraphPad Software, San Diego, CA, USA). *P*-values <0.05 were considered significant.

## 3. Results

### 3.1. Serp-1 Promotes Full Thickness Wound Healing in a Dose-Dependent Manner in Mice 

**Figure 2:**
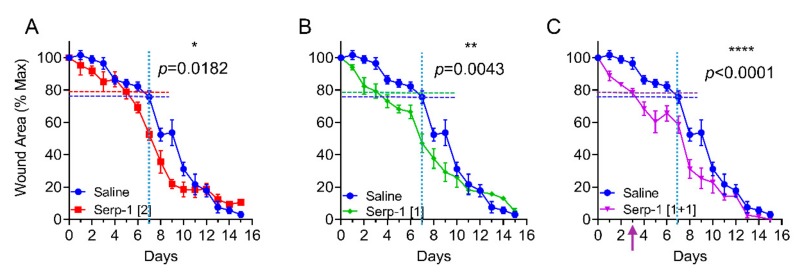
Serp-1 dose- and schedule-dependently accelerates full-thickness wound healing in mice. (A–C) The full course of wound healing for mice with full-thickness wounds treated with saline alone (*N* = 13) or Serp-1 at a dose of (**A**) 2 μg/mouse (Serp-1 [2]; *N* = 6), (**B**) 1 μg/mouse (Serp-1 [1]; *N* = 7), or (**C**) 1 μg/mouse on days 0 and 3 (Serp-1 [1+1]; *N* = 5). Dotted horizontal cross lines indicate equivalent healing of Serp-1 mice on day 3 as is achieved by saline alone on day 7.

We analyzed the effects of Serp-1 on treatment of full thickness wounds in a splinted wound healing model in wild-type C57BL6/J mice, as described in the Experimental Section (Figure 2A,B) [52,53,54]. The initial dose of Serp-1 applied to the wounds was 2 µg/mouse, based on prior intraperitoneal doses of 100 µg/kg/bodyweight used in other models of vascular inflammatory diseases and based on an average mouse weight of 20 g [43]. Healing was most accelerated in the early stages, with 2.0 µg Serp-1 treatment exhibiting approximately 20% closure at day five, which was only achieved by day seven with saline. For mice with 2.0 µg Serp-1 treatments, wounds already had 47% closure at day seven (*p* = 0.015 versus saline at day seven, Figure 2C-1). 

When the Serp-1 dose was reduced to 1.0 μg/wound/mouse we observed a faster wound closure rate. These mice only needed a mean of three days to achieve the same degree of closure (~20%) as the control group at day seven or for the higher dose Serp-1 (2.0 μg) at day five, respectively (Figure 2C-1,C-2; *p* = 0.0182). Compared to the saline group, treatment with 1.0 μg Serp-1 treatment led to wound closure four days earlier in the first week (*p* = 0.0043 versus saline at day seven). Thus, 1.0 µg/wound in a 20 g mouse was a more effective dose than 2.0 µg/wound.

### 3.2. Repeated Dosing Extends the Therapeutic Effects of Serp-1 in Wound Healing

We next tested repeated treatment with the same total amount protein (2 μg) by application of Serp-1 given as a 1.0 μg/20 μL saline/10 mm^2^ wound/mouse at day zero and day three, respectively. Wound healing was further enhanced with 40% wound closure at day five (Figure 2C; *p* < 0.0001), while there was less than 20% wound closure in saline-treated controls (Figure 2A) and only 30% in single dose treatment groups (Figure 2C). Thus, these results demonstrate that the therapeutic efficacy of topical treatment for Serp-1 solution is extended by repeat dosing, further promoting wound healing in the mouse model.

### 3.3. Serp-1 Promotes Wound Closure via Urokinase-Type Plasminogen Activator Receptor (uPAR) 

**Figure 3:**
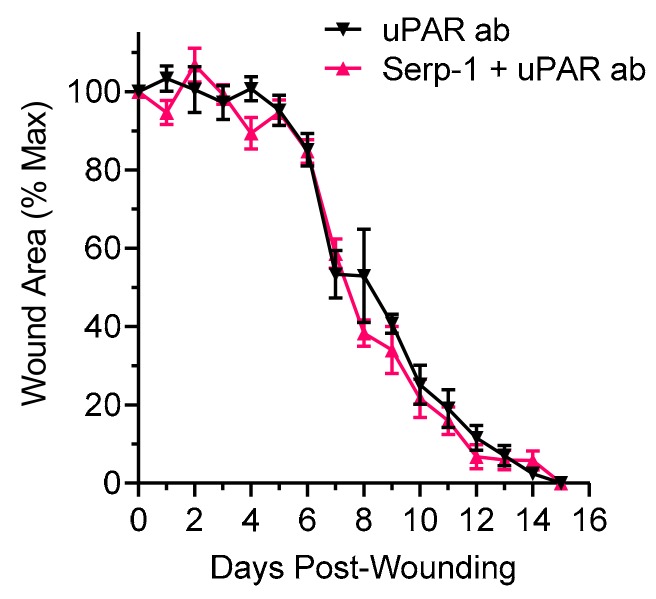
Urokinase-type plasminogen activator (uPAR) neutralization abolishes the wound healing promotion effects of Serp-1. Full thickness wounds of mice treated with uPAR antibody alone (*N* = 5) or Serp-1 with uPAR antibody (*N* = 5) were followed for 15 days with daily planimetric measurements and wound area was determined. Serp-1 dose was 1 µg on day 0 and on day 3, as previously described. Antibody dose was 1 µg on day 0 and on day 3.

In prior work, thrombolytic protease urokinase-type plasminogen activator (uPA) and the uPA receptor (uPAR) were identified as key molecular targets for Serp-1. Serp-1 anti-inflammatory activity is lost in uPAR-deficient aortic allograft transplants [40,46]. We assessed whether Serp-1 therapeutic efficacy in wound healing was uPAR-dependent by antibody neutralization of uPAR in the wound bed. On the day of wounding and on day three post-wounding, uPAR antibody (uPAR ab) was applied to the wound area immediately prior to Serp-1 application. Neutralization of uPAR in the wound bed completely abolished Serp-1 therapeutic benefit as measured by planimetry, demonstrating that Serp-1 functions in an uPAR-dependent manner to promote wound healing (Figure 3; *p* = 0.8710).

### 3.4. Serp-1 Promotes M2 Macrophage Differentiation without Significant Neutrophil Infiltration during Wound Healing

**Figure 4:**
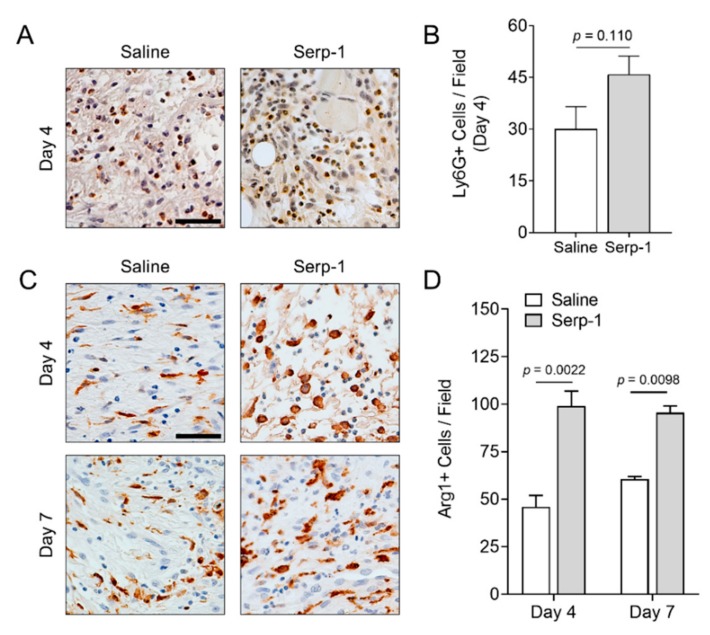
Serp-1 stimulates enhanced anti-inflammatory immune response in wounds. (**A**) Representative immunohistochemistry images of Ly6G staining in 4-day-old wounds of mice treated with saline (*N* = 4) or Serp-1 (*N* = 4). (**B**) Quantification of Ly6G+ cells (representative of peripheral granulocytes). (**C**) Representative immunohistochemistry images of Arg-1 staining in 4- and 7-day-old wounds of mice treated with saline (*N* = 4) or Serp-1 (*N* = 4). (**D**) Quantification of Arg1+ cells (representative of M2-polarized macrophages) at day 4 and day 7. Scale bars represent 50 µm.

We sought to determine the effect of Serp-1 on specific innate immune cell responses during wound healing. Neutrophil infiltration is an early event in wound healing, generally peaking within the first two to four days post-wounding [11,19,63,64]. We analyzed neutrophil infiltration at sites of wound healing with and without treatment with Serp-1 at day four by immunohistochemical staining with antibodies against Ly6G (Figure 4A). Quantification of neutrophil counts at day four found no significant difference between nontreated and Serp-1-treated wounds (*p* = 0.110) (Figure 4B). 

We next measured the presence of M2-polarized anti-inflammatory macrophage cell counts during wound-healing by IHC with antibodies against Arginase-1 (Arg-1), a prototypic M2 marker in mice (Figure 4C) [65,66]. M2 macrophages promote a pro-resolution stage in wound healing [67]. Arg-1 positive macrophages were significantly increased at days four and seven in wounds treated with Serp-1 versus wounds treated with saline (Figure 4D; *p* = 0.0022 at day four and *p* = 0.0098 at day seven). Thus, Serp-1 promotes an anti-inflammatory environment in full thickness wounds with minimal effects on neutrophil responses.

### 3.5. Topical Serp-1 Promotes Vascularization during Wound Healing

**Figure 5:**
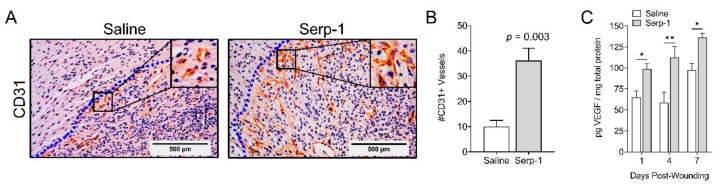
Topical delivery of Serp-1 enhances periwound vascularization. (**A**) Representative images of immunohistochemistry of wounds at day 7 probed with anti-CD31 antibody. Inset highlights CD31+ endothelial cells in periwound blood vessels. Dotted blue line indicates the wound boundary, with the wound bed to the left of the line. (**B**) Quantification of CD31-positive vessels at day 7 in the periwound area of mice treated with saline (*N* = 4) or Serp-1 (*N* = 4). Bars represent mean ± standard error. Statistics were calculated by Student’s T-test. (**C**) Quantification of VEGF in wound tissue treated with saline or with Serp-1 (1 µg + 1 µg schedule) on days 1, 4, and 7 post-wounding procedure (saline *N* = 3, Serp-1 *N* = 4 for all time points). VEGF, Vascular endothelial growth factor. Statistics were calculated by two-way ANOVA with Fisher’s LSD. *p < 0.05, **p < 0.01.

We next examined new vessel growth (vascularization) at seven days post-wounding—an intermediate time during which wounds were observed to begin healing at a rapid rate (Figure 2C). We analyzed vascularization by immunohistochemistry with antibodies against the endothelial marker CD31 (also called PECAM-1), a marker for endothelial cells commonly used to indicate wound bed blood vessels [68]. The number of positive staining vessels in different optical fields was quantified from four pairs of saline- and Serp-1-treated wounds. We first noted that Serp-1-treated mice had appreciably more CD31-positive vessels, with increased length and thickness, versus mice treated with saline alone (Figure 5A). Quantitatively, vessel density was significantly increased to 36.3 ± 4.9 in the peri-wound area of mice treated with Serp-1 versus 10.0 ± 2.5 in the saline control mice (*p* = 0.003; Figure 5B). These observations are further supported by a significant Serp-1-induced increase in VEGF in the wound beds at days 1, 4 and 7 (Figure 5C).

### 3.6. Serp-1 Treatment Improves Collagen Organization at Sites of Scar Formation

**Figure 6:**
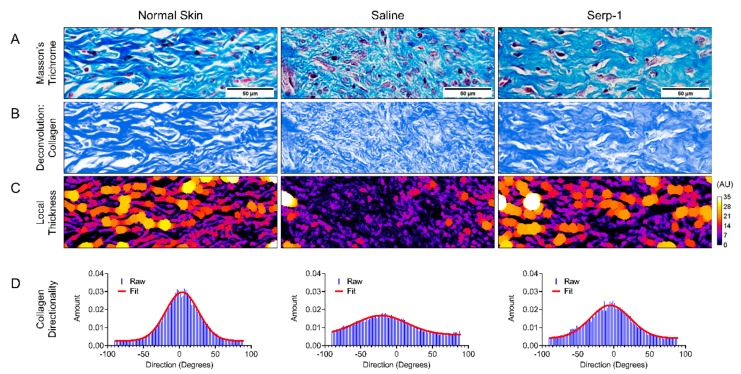
Quantitative collagen texture analysis. (**A**) Representative 20× images of normal (left), saline treated (middle), and Serp-1 treated (right) skin stained with Masson’s trichrome. (**B**) Deconvolution of the blue-stained connective tissue component of the trichrome staining. (**C**) Local thickness analysis with heat-mapped visualization (more purple/black = less thick; more yellow/white = more thick). (**D**) Raw (blue) and fit (red) data of directionality of the collagen component of the trichrome stain. Greater peaks in the data indicate more preference for a single direction by the component of the image, while a more flat distribution indicates less directionality or preference for a single direction.

**Figure 7:**
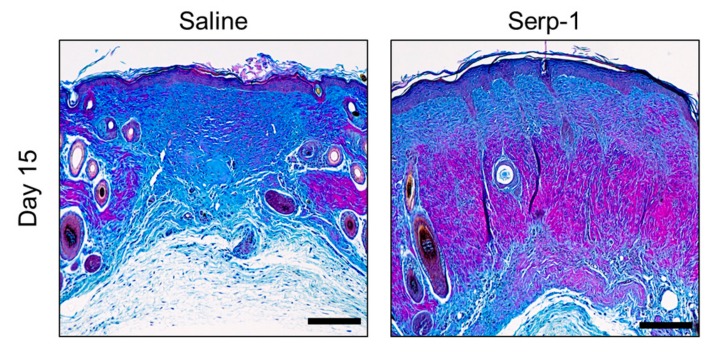
Serp-1 treatment promotes collagen maturation in wounds. Representative images of Herovici’s polychrome staining of wound tissue at day 15. Blue stain indicates younger collagen (Type 3); Red/pink stain indicates mature collagen (Type 1). Scale bars represent 200 µm.

During wound healing, collagen accumulation and organization correlate closely with scar formation. We performed Masson’s trichrome staining (Figure 6A) and Herovici’s polychrome staining (Figure 7) to study the dermal collagen fibers after 15 days of wound healing. Local scar thickness (Figure 6C) and directionality (Figure 6D) of collagen in scars were analyzed using ImageJ/FIJI after Masson’s trichrome staining under microscopic examination (20× magnification). With Serp-1 treatment, collagen fibers demonstrated increased bundle thickness as well as more organized directionality, with close similarity to the features found in the collagen network of normal skin. Conversely, skin of mice treated with saline alone demonstrated limited maturation, as indicated by small bundle thickness, as well as reduced directionality, indicative of poorly organized collagen deposition and characteristic of scar architecture [69,70]. 

We sought to further investigate the quality of the collagen in healed wounds by Herovici’s staining, a specialized method to differentiate between young (Type 3, more blue) and mature (Type 1, more red/pink) collagen bundles [57,71]. Whereas saline-treated wounds displayed primarily a blueish hue in the connective tissue by Herovici’s stain, Serp-1-treated wounds were substantially more reddish hued, indicating a greater degree of maturation in the collagen bundles of Serp-1-treated mice (Figure 7). Taken together, these data demonstrate that in addition to more rapid wound healing, the quality of the healed wound site is improved versus saline treatment and is more similar to that of normal, unwounded skin. 

### 3.7. Single Application of Serp-1 via Chitosan-Collagen Hydrogel Promotes Wound Healing in a Mouse Model

**Figure 8:**
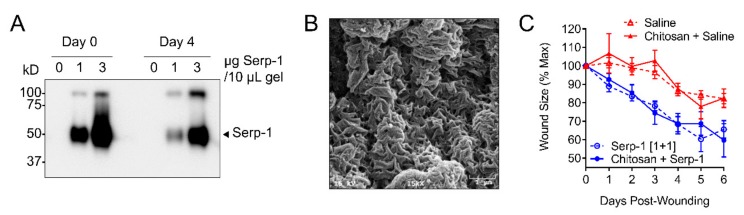
Application of chitosan-collagen hydrogel containing Serp-1 can efficiently promote wound healing in a mouse model. (**A**) Immunoblot demonstrating dose-dependent release of Serp-1 from the chitosan-collagen hydrogel into phosphate-buffered saline over a period of 4 days. Arrow indicates monomeric Serp-1, high molecular weight band represents Serp-1 dimers. (**B**) Scanning electron microscope imaging at 15,000× magnification of chitosan-collagen hydrogel illustrates a complex, porous, and regularly folded surface. (**C**) Time course demonstrating that saline alone (*N* = 4; red dotted), hydrogel loaded with saline (*N* = 4; red solid), Serp-1 [1+1] (*N* = 4; blue dotted), and hydrogel loaded with Serp-1 (*N* = 4; blue solid) have identical wound healing kinetics.

A major goal for wound healing treatments is to reduce further damage to new tissue growth through invasive treatments causing secondary injury. Repeat administrations or injections of treatments can cause further damage. To address this need, we developed a non-covalently crosslinked composition of chitosan and collagen as a carrier for sustained functional delivery of Serp-1 directly to the wound bed. When prepared, the homogenously mixed type I collagen solution and Serp-1-bound chitosan form a translucent, spreadable gel (Figure 8A). We verified the dose-dependent, sustained release of Serp-1 from the chitosan-collagen hydrogel into aqueous solution in vitro with minimal dimerization (Figure 8B). Ultrastructural analysis of dehydrated hydrogel by scanning electron microscopy revealed a highly complex and porous appearance, with many regularly dispersed folds, indicating that the hydrated hydrogel has a high capacity surface area for potential protein binding (Figure 8C).

After demonstrating that delivery of stable, monomeric Serp-1 protein was sustained by the chitosan-collagen hydrogel release in vitro, we subsequently tested the therapeutic efficacy in the mouse dorsal wound model by topical application of 30 μL of gel containing 3.0 μg Serp-1 onto the wound site at day zero (Figure 8D). The gel was directly loaded onto the wound surface with a sterile micropipette tip and covered by silicon splint and Tegaderm (see Materials and Method). Compared to wounds treated with saline or chitosan only, wounds treated with Serp-1 gel had similar closure rates as for wounds treated with 1.0 µg of Serp-1 followed by a bolus at day three (Figure 8E). These data indicate that sustained, therapeutically effective release of Serp-1 by chitosan-collagen hydrogel after a one-time application is capable of significantly promoting wound healing. 

## 4. Discussion

Healing of large wounds remains an important clinical challenge for numerous demographics, including soldiers and surgical patients, as well as populations with complex comorbidities such as diabetics and the elderly. Attention has thus increasingly turned towards developing new treatments for rapidly restoring the skin barrier and reducing scarring in wounds [11,72]. In this study, we tested the therapeutic efficacy of a purified Myxoma virus-derived Serp-1 protein biologic in a full thickness wound healing model in mice.

In an initial analysis, Serp-1 was applied as a saline solution given directly to the wound bed. Daily planimetric measurements of the wound area revealed that Serp-1 dose-dependently accelerated wound closure when compared to saline control treatments. Interestingly, 1) the most significant acceleration was observed during the earliest stages of wound healing, and 2) the lower dose of Serp-1 (1.0 µg/wound) was more effective than the higher dose (2.0 µg/wound). The finding of early acceleration is an important clinical consideration, given the fact that the first phase of dermal wound healing is crucial to protect against infection and further damage [22]. The second observation that the lower dose was more effective than the higher dose speaks to the need to modulate, not shut down, the immune response in wound healing. Indeed, immune response is well established as an important factor of healthy wound healing.

We hypothesized that the efficacy of Serp-1 would diminish over time due to metabolic turnover and thus tested multiple doses for treatment [51]. Repeated administration of 1.0 µg Serp-1 in saline solution was found to be more effective than a single dose, indicating that the duration of effectiveness could be extended by lengthening the dose period. A risk factor with multi-dose treatments in dermal wounds is the possibility of secondary injury, which may prolong the healing time. To avoid the need to access the wound for repeated administration, we developed a biocompatible hydrogel composed of chitosan and collagen, a matrix that has previously proven successful in wound dressing development [73]. By loading the chitosan-collagen hydrogel with Serp-1, we found wound healing was accelerated to the same degree as with repeated administration at the equivalent dose, but without the need to re-access the wound. Thus, when delivered in a biocompatible chitosan-collagen hydrogel matrix, Serp-1 provides a sustained and durable promotion of wound healing with a reduction in the risk of secondary injury.

Prior work indicated that Serp-1 interacts with proteins of the thrombotic and thrombolytic cascades, as well as secondary participants such as filamin B, vitronectin, and the urokinase-type plasminogen activator receptor (uPAR) [45,46]. Specifically, inflammatory cell infiltration was found to be dependent on uPAR in in vitro studies [46]. uPAR is enriched at the leading edge of invading pro-inflammatory macrophage at sites of arterial injury and in healing dermal wounds [74] and is proposed to play a critical role in regulating the immune cell activity in healing wounds [75]. We thus investigated if Serp-1 treatment efficacy was dependent on uPAR. Neutralization of uPAR in the wound bed by topical application of a monoclonal anti-uPAR antibody completely nullified the efficacy of Serp-1. Treatment with uPAR antibody alone or uPAR antibody given together with Serp-1 treatment resulted in identical healing curves indistinguishable from treatment with saline alone. These results indicate that the ability of Serp-1 to promote wound healing is dependent on uPAR. The precise molecular pathways for interaction between Serp-1 and uPAR (i.e., inhibition, stabilization, or activation) remain to be explored. Furthermore, the downstream consequences of the interaction between Serp-1 and uPAR on pathways known to be affected by the plasminogen activation system in wound healing (e.g., SMAD and TGFβ signaling [76]) are open questions.

To better understand the Serp-1-mediated effects on immune cell dynamics in the healing wound, we stained for macrophages and neutrophils. These cells are key drivers in the early stages of wound healing, and this was observed as the time during which Serp-1 had the greatest impact in Serp-1-mediated healing acceleration. Immunohistochemistry was performed using prototypical markers of pro-resolution M2 macrophages (Arginase-1) and neutrophils (Ly6G). Quantitation of stained cells demonstrated that Serp-1 treatment increased M2 macrophages with no significant effect on neutrophil dynamics. It should be noted that numerous, more detailed polarization schemes have been proposed, including M2a-c, M(IL4), M(IL10), and others [77,78]. High Arg-1-expressing cells commonly fall into the category of M(IL4) and M2a, depending on the selected classification scheme. Thus, Serp-1 produces a pro-resolution environment in the wound bed.

New blood vessel formation (i.e., vascularization) is also a critical component of wound healing. Thus, numerous strategies have been developed to promote increased vascularization at sites of skin wounds [79,80]. The thrombolytic proteases, uPA and tPA, have been associated with growth factor release and mammalian serpins such as plasminogen activator inhibitor-1 (PAI-1) and have been reported to alter angiogenesis. Based upon known canonical targets overlapping with PAI-1, we hypothesized that Serp-1 has the potential to alter vessel growth in wound healing. Serp-1 treatment increased periwound angiogenesis with significantly more CD31-positive vessels in the healing wound. Furthermore, the CD31-positive vessels are appreciably thicker and longer, which we have observed in many sections and cannot attribute to a sectioning artifact alone. The increased angiogenesis in Serp-1-treated wounds was supported by a significant increase in VEGF in the wound beds, in agreement with the well-established role of VEGF in regulating vessel formation [81].

Collagen deposition and maturation play a central role in angiogenesis during tissue regeneration as well as wound remodeling and scarring. Collagen I is an important factor in angiogenic sprouting and is associated with increased and improved vessel formation [82]. In contrast, increased deposition of collagen III reduces the density of blood vessels at sites of wound healing [83,84]. Here, we have further demonstrated that Serp-1 treatment is associated with more organized collagen deposition (Masson’s trichrome), as well as more collagen maturation (Herovici’s polychrome). Thus, Serp-1-treated wounds not only heal faster, but also have improved remodeling.

## 5. Conclusions

We report here that treatment with Serp-1, a purified myxoma virus-derived SERPIN and immune modulating protein biologic, accelerates full thickness wound healing in wild-type mice. Serp-1 treatment is associated with a pro-resolution immune phenotype, increased angiogenesis, and improved remodeling. Furthermore, we demonstrated that Serp-1 can be delivered in a durable and sustained manner by release from a biocompatible chitosan-collagen hydrogel, reducing the risk of secondary injury by repeated administration of treatments to the healing wound. Future work will focus on testing Serp-1 in complex wounds with comorbidities (e.g., infection, diabetes, burns), delineation of the molecular mechanism associated with Serp-1 therapeutic function (e.g., induction of cytokines and chemokines in the wound bed), and defining the mechanism of uPAR-dependent efficacy. Furthermore, prior clinical trials have demonstrated the remarkable safety profile of Serp-1 in humans [44], and thus, future clinical testing of Serp-1 in cutaneous simple and complex wounds may be of interest. In conclusion, multipotent and cross-species virus-derived immune modulators, protein biologics, are a highly promising approach to control and improve the healing process and clinical wound management.

## Supplementary Materials

The following are available online at https://www.mdpi.com/2077-0383/8/10/1626/s1, Figure S1: Full-thickness wounding in the mouse model.
