# Supplementary material for: A Virus-Derived Immune Modulating Serpin Accelerates Wound Closure with Improved Collagen Remodeling
Source: J Clin Med. 2019 Oct 4;8(10):1626. doi: 10.3390/jcm8101626 (PMC6832452; doi:10.3390/jcm8101626)
Supplement: Supplementary file 1 [file jcm-08-01626-s001.pdf]

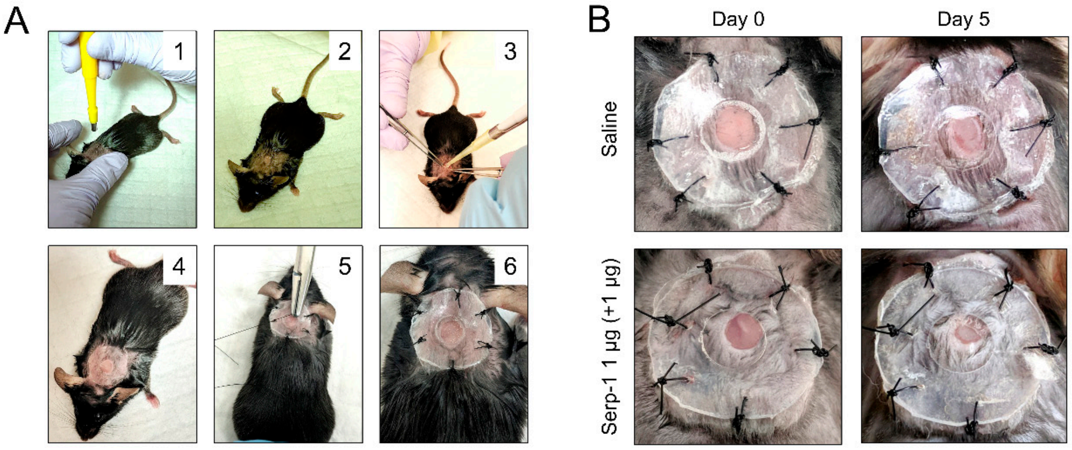

Figure S1. Full-thickness wounding in the mouse model. (**A**) Overview of the surgical method for creating the full-thickness splinted wound healing model: (1,2) a punch biopsy injury was performed on the dorsal, interscapular skin of an anesthetized mouse; (3) Serp-1 treatment or saline control was delivered directly to the wound bed; (4) a silicone splint overlaid with Tegaderm was applied to the back with a thin layer of cyanoacrylate glue; (5) the silicone splint was sutured to the back of the mouse; (6) the wound was photographed and the mouse was monitored until fully recovered. (**B**) Representative examples of day 0 and day 5 wounds of mice treated with saline alone (top panels) or Serp-1 as a 1 μg/mouse dose on both day 0 and day 3 post-wounding.
